# Effect of Dietary Inclusion of Riboflavin on Growth, Nutrient Digestibility and Ruminal Fermentation in Hu Lambs

**DOI:** 10.3390/ani13010026

**Published:** 2022-12-21

**Authors:** Na Ren, Xuanzi Zhang, Xiaoyan Hao, Yingrui Dong, Xinggang Wang, Jianxin Zhang

**Affiliations:** College of Animal Sciences, Shanxi Agricultural University, Jinzhong 030801, China

**Keywords:** riboflavin, rumen fermentation, nutrient digestibility, microbiota, Hu lambs

## Abstract

**Simple Summary:**

Riboflavin (RF), in the form of flavin mononucleotides and flavin adenine dinucleotides, is involved in the transfer of electrons during redox reactions and plays an important role in animal metabolism and growth. Dietary RF supply has the potential to improve growth performance and nutrient digestion in lambs, and this might be associated with the stimulatory impacts of RF on ruminal microbial growth. The effects of RF on lamb growth performance and rumen fermentation were evaluated. The results showed that RF supplementation improved the growth performance, nutrient digestion and rumen fermentation in lambs.

**Abstract:**

The study evaluated the influences of riboflavin (RF) supply on the growth performance, nutrient digestibility and ruminal fermentation in lambs. Forty-eight Hu lambs were randomly assigned into four groups receiving RF of 0, 15, 30 and 45 mg/kg dry mater (DM), respectively. Increasing RF supply did not affect the DM intake, but quadratically increased the average daily gain and linearly decreased feed conversion ratio. Total-tract DM, neutral detergent fibre, acid detergent fibre and crude protein digestibility increased quadratically. Rumen pH and propionate molar percentage decreased linearly, total volatile fatty acids concentration, acetate proportion and the ratio of acetate to propionate increased linearly, but ammonia nitrogen concentration was unchanged with increasing RF supply. Linear increases were observed on the activities of carboxymethyl-cellulase, xylanase, pectinase and protease, and the populations of bacteria, fungi, protozoa, dominant cellulolytic bacteria, *Ruminobacter amylophilus* and *Prevotella ruminicola*. Methanogens population was not affected by RF supplementation. The microbial protein amount and urinary total purine derivatives excretion increased quadratically. The results indicated that 30 mg/kg DM RF supply improved growth performance, rumen fermentation and nutrient digestion in lambs.

## 1. Introduction

Riboflavin (RF) functions in the form of flavin adenine dinucleotide (FAD) and flavin mononucleotide (FMN) [1,2]. Both FAD and FMN are the prosthetic groups of flavoproteins that participate in various electron-transferring reactions in ATP generation, electron-scavenging and biosynthetic pathways [3]. It has been demonstrated that energy metabolism efficiency was improved with dietary RF inclusion in mice [4] and humans [5]. The RF was an essential nutrient for both ruminants and rumen microorganisms [2]. Early in vitro studies noted that RF supplementation increased the numbers of *Ruminococcus flavefaciens* and *R. albus* in ruminal fluid [6], and that the growth of ruminal protozoa was stimulated with B vitamins containing an addition of RF [7]. A recent study in bulls found that dietary supplementation with RF tended to increase average daily gain (ADG), total-tract nutrient digestibility, and ruminal total volatile fatty acids (VFA) concentration, as well as fungi, protozoa, and bacteria populations [8]. Some B vitamins supplied by cross-feeding among microbes were not sufficient to sustain some species of bacteria growth in the rumen [9]. Approximately 99.3% of dietary supplemented RF disappeared in the rumen of cows [10], and that RF should be utilized by microorganisms to maintain their growth. Furthermore, dietary folic acid or niacin inclusion improved growth performance in lambs [11,12]. These results indicated that RF derived from ruminal bacteria synthesis and diets could not meet the requirements of ruminants and ruminal microbes.

The present study was designed based on the hypothesis that dietary RF supply was required for ruminal microbes and lambs per se. Therefore, the study investigated the effects of RF addition on growth performance, ruminal fermentation, and nutrient digestion in lambs.

## 2. Materials and Methods

### 2.1. Lambs, Expeirmental Design and Diets

This study was authorised by the Animal Care and Ethical Committee of Shanxi Agricultural University. Forty-eight purebred Hu lambs, 90 ± 10 days of age and 18.8 ± 0.83 kg of body weight (BW), were randomly assigned to four treatments: control, low-riboflavin (LRF), medium-riboflavin (MRF) and high-riboflavin (HRF) with RF of 0, 15, 30 and 45 mg/kg DM supplementation, respectively. The supplementation level of RF (feed grade, 980 mg RF/g, Beijing Solarbio Biotechnology Co., Ltd., Beijing, China) was determined according to the results (0.8 mg/kg BW) of Wu et al. [8] in bulls. Supplementary RF was mixed into the premix, and then into the basal diet. The lamb diet was formulated based on the NRC [13] recommendations (Table 1) and was fed as a whole mixed pellet diet (4 mm diameter). The content of RF in the basal diet was measured using an approach described by Santschi et al. [10], and was 6.1 mg/kg DM. The experiment period lasted for 85 days; 15 days were used for lamb adaptation and 70 days for collecting samples. The lambs were individually kept in a 3 × 0.8 m stall, fed twice daily at 5:30 and 17:30, and consumed water and diets freely.

### 2.2. Data and Sample Collection

During the sampling period, each lamb was weighed on days 0, 30, and 60 before the morning feeding. The individual DM intake (DMI) was calculated based on the difference between the daily feed offered and orts. On day 61, all lambs were individually housed in a metabolic crate (120 × 75 cm) equipped with a leaky floor. The faecal samples were collected through a nylon sieve plate under the metabolic crates, and urine samples were collected through a funnel into a bucket with 100 mL of 10% (*w/w*) H_2_SO_4_ [14]. On days 66 through 70, feed offered and refused were sampled for each lamb. Daily total faecal and urine production of each lamb were quantified at 6:00. The faecal and urine samples that were representative of 10% of the daily faecal and urine production were collected. The samples of feed, refusals, faeces, and urine were stored at −20 °C, and mixed by lamb, respectively, at the end of the trial. Feed and faecal samples were dried at 65 °C to achieve a constant weight, and ground through a 1-mm screen (SF130B, Tianhe Machinery Instrument Co., Ltd., Shanghai, China).

On days 69 and 70, ruminal fluid samples were collected at 5:00, 8:00, 11:00, and 14:00 using a stomach tube. The initial 80 mL of samples collected were discarded to avoid the contamination of saliva, and the subsequent 80 mL were retained. After the pH was determined, the samples were filtered through nylon cloth. Two 5 mL aliquots of ruminal fluid were stored at −20 °C for VFA and ammonia nitrogen determination. Two 10 mL ruminal fluid filtrates were frozen at −80 °C for enzyme activities measurement and microorganism DNA extraction.

### 2.3. Chemical Analyses

Feed and faeces samples were determined for DM (AOAC, 2005 [15]; method 930.15) by oven-drying for 3.5 h at 105 °C, Ash (AOAC, 2005 [15]; method 942.05) by combustion in a muffle furnace for 3 h at 550 °C, nitrogen (AOAC, 2005 [15]; method 984.13) by a Kjeltec Auto Analyzer (K2300, Tianjin Humanda Technology Development Co., Ltd., Tianjin, China), and neutral detergent fibre (NDF) [16] and ADF (AOAC, 2005 [15]; method 973.18) by using an F800 fiber analyzer (Hanon Advanced Technology Group Co., Ltd., Jinan, China). The content of organic matter (OM) was calculated by the difference between DM and ash. The total tract nutrient digestibility was evaluated as: (nutrient intake—nutrient in feces)/nutrient intake.

Ruminal VFA was determined via gas chromatography (GC8950, Tuming Optical Instrument Co., Ltd., Shanghai, China). Ruminal ammonia nitrogen concentration was determined by a UV spectrophotometer (RM-7230G, Qingdao Ruiming Instrument Co., Ltd., Qingdao, China) based on AOAC [15]. Activities of α-amylase, protease and fibrolytic enzymes were measured using the procedures described by Agarwal et al. [17], and were expressed as reducing sugars content released per mL of ruminal fluid over a 30-min incubation at 39 °C.

Urinary allantoin, xanthine, and hypoxanthine levels were tested based on the procedures of Chen and Gomes [18], and uric acid level was measured by the uric acid kit (Shanghai Enzyme Link Biotechnology Co., Ltd., Shanghai, China). The amount of rumen microbial protein (MCP) was calculated from urinary purine derivatives according to the reports of Chen and Gomes [18].

### 2.4. Extraction of Microbial DNA and Real Time-PCR

The total microbial DNA was isolated from 1.0 mL of rumen fluid sample by using the repeated bead-beating plus column (RBB + C) method, as described by Yu and Morrison [19], the DNA concentration was measured using a nucleic acid protein assay, and the DNA concentration was diluted to 100 ng/mL using TE buffer. The target-specific microbial primers set sequences are shown in Table 2. Based on the microbial DNA treatment pool, ten standards were derived by using the conventional PCR. Subsequently, PureLink Quick Gel Extraction and PCR Purification Combo Kit (Thermo Fisher Scientific Co., Ltd., Shanghai, China) were used to purify amplified products, which were quantified using a spectrophotometer. The copy counts of the standards for each bacterium was determined based on the length and mass concentration for amplification products. The target DNA was quantified from 10^1^ to 10^8^ DNA copies using a 10-fold serial dilution method [20]. Standard DNA samples were prepared for each qPCR assay, and the target DNA was quantified according to the TaKaRa Reagents SYBR ^®^ Primic Ex TaqTM (Tli RNaseH Plus) kit instructions using a StepOne ™ system real-time fluorescent quantitative PCR instrument (ABI StepOnePlus). The PCR reaction system (20.0 μL) consisted of 10.0 μL of SYBR ^®^ PrimicExTaqTM (Tli RNaseH Plus) (2×), 2.0 μL of DNA template, 0.8 μL of each primer (10 μM/μL), 0.4 μL ROX Reference Dye (50×) * 2 and 6.0 μL nuclease-free water. PCR reactions were 95 °C denaturation for 1 min; 95 °C for 15 s, 60 °C for 30 s, 40 cycles; 95 °C for 15 s, 60 °C for 1 min, 95 °C for 15 s for 40 cycles.

### 2.5. Data Statistics and Analysis

The data obtained from the experiment were analyzed by using SPSS software version 22.0 [21] with one-way ANOVA of the generalized linear model (GLM), where dietary concentration of RF represented the treatment effect. Individual lambs were regarded as experimental units. In addition, multiple comparisons were performed by Duncan’s method when differences were significant, and linear and quadratic effects were analyzed using orthogonal polynomials. *p* < 0.05 indicates significant differences, and 0.05 < *p* < 0.1 indicates a trend.

## 3. Results

### 3.1. Growth Performance

Dietary RF inclusion did not influence DMI in lambs (Table 3). The initial and final BW of lambs were similar among treatments. With increasing the level of RF supply, ADG quadratically increased (*p* = 0.023) and was higher (*p* = 0.037) for lambs in the MRF than for those in the HRF and control. Feed conversion ratio (FCR) linearly decreased (*p* = 0.026) and was lower (*p* = 0.019) for MRF compared with the control.

### 3.2. Nutrient Apparent Digestibility and Rumen Fermentation

Increasing RF supplementation, nutrient apparent digestibility responded quadratically (*p* < 0.05) (Table 4). The apparent digestibility of total-tract DM, OM, CP and NDF was higher (*p* < 0.05) for MRF compared with the control and HRF, and that of ADF was greater (*p* = 0.022) for lambs receiving RF 30 mg/kg DM supply compared with those in LRF, HRF, and the control. By increasing dietary RF supply, ruminal pH and propionate molar percentage decreased linearly (*p* = 0.032); total VFA content, percentages of acetate and isobutyrate, and ratio of acetate to propionate linearly increased (*p* < 0.050); but valerate, butyrate and isovalerate proportions were not influenced. Ruminal pH in lambs receiving 30 mg/kg DM RF was lower (*p* = 0.014) than in animals receiving the control diet. Ruminal total VFA content was highest in MRF, followed by LRF and HRF, and was lowest in the control. The proportion of acetate was greater (*p* = 0.002), and propionate was lower (*p* = 0.030) for lambs receiving the RF supply. Hence, the acetate to propionate ratio was highest in LRF and MRF, intermediate in HRF and lowest in the control. Ruminal isobutyrate percentage was highest in MRF, followed by LRF and HRF, and lowest in the control.

### 3.3. Ruminal Microbial Enzyme Activity and Microbial Number

The activities of protease, pectinase, carboxymethyl-cellulase and xylanase responded linearly (*p* < 0.05), but α-amylase and cellobiose were unaltered with increasing RF supplementation (Table 5). The protease, pectinase, and carboxymethyl-cellulase activity values were higher (*p* < 0.05) for MRF compared to the control. The xylanase activity was greater (*p* = 0.038) for MRF and HRF than for the control. The numbers of dominant cellulolytic and amylolytic bacteria, total bacteria, fungi, and protozoa linearly increased (*p* < 0.05), but the methanogens population was unchanged with RF supply. The numbers of total bacteria, *R. flavefaciens*, *R. albus*, *F. succinogenes*, *Rb. amylophilus* and *P. ruminicola* were greater (*p* < 0.05) in lambs receiving 30 mg/kg DM RF than in lambs receiving the control diet. The population of total fungi was highest in MRF, followed by LRF and HRF, and then the control. The numbers of protozoa and *B. fibrisolvens* were higher (*p* < 0.05) for MRF than for LRF and the control.

### 3.4. Urinary Purine Derivatives and MCP

Urinary allantoin, xanthine and hypoxanthine, total purine derivatives (TPD), and microbial protein (MCP) levels increased quadratically (*p* < 0.05) with elevating the addition level of RF (Table 6). The xanthine and hypoxanthine, TPD and MCP in urine were greater (*p* < 0.05) for MRF compared with the control and HRF, and allantoin was higher (*p* = 0.012) for MRF than for other groups. The excretion of uric acid was unchanged with RF supply.

## 4. Discussion

### 4.1. Growth Performance

The unchanged DMI with dietary RF supply agreed with the result of Wu et al. [8] in bulls. The increase in ADG might be due to the increase in nutrient apparent digestibility and rumen total VFA concentration. The results showed that nutrient utilization efficiency was improved by RF supply, as reflected by the decrease in FCR. In addition, FAD and FMN participated in electrons transfer and ATP generation in cells [22,23]. It has been demonstrated that the efficiency of energy metabolism increased with RF supplementation in mice [4] and humans [5]. The positive effect of RF on energy utilization efficiency might also have contributed to the increase in ADG in lambs. Likewise, Wu et al. [8] reported that dietary RF inclusion improved ADG and FCR in bulls. However, ADG in lambs receiving HRF addition diets was lower compared with those consuming MRF diets and was similar with those in the control. The changes in ADG were consistent with responses of total-tract nutrient digestibility and rumen total VFA concentration and fungi population, which were lower for HRF than for MRF addition. Similarly, study of bulls found that rumen total VFA concentration and NDF degradability were lower for dietary 900 mg RF/d addition compared to 600 mg RF/d [8]. These results suggested that a higher level of RF in the rumen might interfere with microbial growth or metabolism, causing a decrease in nutrient digestion and ADG.

### 4.2. Nutrient Apparent Digestibility and Rumen Fermentation

That RF supply increased DM, OM, NDF, and ADF digestibility in the total-tract was consistent with the increase in rumen total VFA concentration and acetate proportion suggested that dietary RF supply enhanced ruminal nutrient degradation. Study in bulls showed that ruminal DM and NDF effective degradability increased with RF addition [8]. In addition, RF participates in the division and differentiation of cells, and is necessary in keeping normal structure and function of the digestive tract [23,24]. Therefore, dietary RF supply might improve nutrient digestion in the intestine, and this was also a reason for the increase in apparent digestibility of nutrients.

The observed reduction in rumen pH was consistent with the increase in total VFA concentration with dietary RF supply. The accumulation of VFAs caused pH decrease [25]. However, the value of pH ranged from 6.24 to 6.51, and was appropriate for microbial metabolism and nutrient degradation [26]. The increase in ruminal total VFA concentration and acetate molar proportion were related to the increase in activities of xylanase, carboxymethyl-cellulase and pectinase, and were in accordance with the increase in total-tract NDF and ADF digestibility. Dietary fibre was hydrolyzed to acetate in the action of cellulolytic enzymes in the rumen [27]. Rumen propionate molar percentage decreased with RF addition, but propionate concentration (23.3, 24.6, 25.7, and 24.9 mM for the control, LRF, MRF, and HRF, respectively) had a tendency towards increase due to the increase in total VFA concentration. The results were consistent with the changes of α-amylase activity with RF supplementation. The increased acetate percentage and reduced propionate percentage led to the increase in acetate to propionate ratio, suggesting that the ruminal fermentation mode was shifted to yield more acetate. Likewise, study of bulls observed increased rumen total VFA concentration and acetate to propionate ratio with dietary RF supply [8]. The increase in isobutyrate proportion was likely due to the increase in protease activity and was a reason for the observed increase in numbers of cellulolytic bacteria (*B. fibrisolvens*, *R. albus*, *F. succinogenes* and *R. flavefaciens*) with dietary RF supply. Ruminal isobutyrate is derived from valine degradation and is a growth factor of cellulolytic bacteria [28]. Previous study of steers indicated that isobutyrate addition increased rumen cellulolytic bacteria relative abundance [29]. The limited response of ruminal ammonia-N concentration to RF supplementation was inconsistent with the observed increases in protease activity and protozoa population and was likely related to the increase in cellulolytic bacteria numbers and MCP synthesis. Rumen protease degrades feed CP and produces ammonia-N [30]. The process of protozoa degrading bacteria also produces ammonia-N [7]. Nevertheless, bacteria, especially fibrolytic bacteria, utilize ammonia-N to synthesize their protein [31].

### 4.3. Ruminal Enzyme Activity and Microbial Number

The observed increase in activities of carboxymethyl-cellulase, xylanase, and pectinase were related to the increase in populations of fungi, protozoa, and fibrolytic bacteria (*R. flavefaciens*, *F. succinogenes*, *R. albus and B. fibrisolvens*), indicating that cellulolytic microbial growth was stimulated by RF supply. The fibrolytic bacteria accompanied by anaerobic fungi and protozoa produce fibrolytic enzymes to ferment fiber to acetate [32,33]. Anaerobic fungi can penetrate plant cell walls, facilitating fibre degradation [34]. The protozoa were responsible for approximately one third of fibre digestion in the rumen [35]. The increase in protease activity was in line with the increase in numbers of *P. ruminicola, B. fibrisolvens,* and *Rb. amylophilus*, which are the predominant bacteria species responsible for degrading protein [36]. The current data indicated that a dietary RF supply was required for ruminal microbial growth in lambs. The FAD and FMN participate in electron transport processes of protein folding, cell signalling, and metabolism of carbohydrates, lipid and amino acids, and play a major role in cellular growth and proliferation [1,37]. Similarly, early in vitro studies observed that supplementation with RF stimulated the proliferation of certain *R. albus* strains [6], and that the survival time of protozoa was prolonged by B vitamins containing the addition of RF [7]. Studies of bulls observed that fibrolytic enzyme activity and populations of fungi, protozoa, and bacteria increased with dietary RF supplementation [8].

### 4.4. Urinary Purine Derivatives and MCP

The increase in urinary TPD excretion and rumen MCP synthesis with RF addition were associated with the increase in ruminal total VFA concentration and bacteria population. The results were consistent with the increase in total-tract CP digestibility and supported the improvement in ADG with RF addition. Rumen VFA are the sources of carbon skeleton and energy in the process of MCP synthesis [38]. Furthermore, rumen ammonia-N concentration ranged from 9.00 to 10.25 mg; 100 mL^−1^ was optimum for MCP synthesis [39]. Likewise, Wu et al. [8] found that RF supply increased urinary total PD excretion in bulls.

## 5. Conclusions

Supplementation with RF 30 mg/kg DM improved growth performance and nutrient digestion in lambs, and these were associated with the stimulatory impacts of RF on rumen microbial growth and protein synthesis. Addition of RF stimulated rumen microbial growth, especially those responsible for fibre degradation, causing the rumen fermentation mode change to produce acetate. However, higher levels of RF (45 mg/kg DM) addition had no influence on ADG and FCR in lambs.

## Figures and Tables

**Table 1:** Ingredient and chemical composition of the basic diet.

Ingredients	Contents (g/kg DM)
Corn straw	250
Peanut shell powder	80
Sunflower leather powder	70
Corn grain, ground	220
Soybean meal	70
Cottonseed meal	60
Corn germ meal	100
Sprayed corn husk	50
Rice bran	50
Calcium carbonate	13
Salt	5
Calcium phosphate	4
Mineral and vitamin premix *	28
Chemical composition	
Organic matter	907.0
Crude protein	133.9
Ether extract	28.0
Neutral detergent fibre	478.3
Acid detergent fibre	221.9
Non-fibre carbohydrate ^1^	266.9
Calcium	6.2
Phosphorus	4.2

* Contained per kg premix: 55 mg Fe, 14 mg Cu, 38 mg Mn, 0.4 mg I, 0.1 mg Co, 18,000 IU vitamin A, 3500 IU vitamin D and 420 IU vitamin E. ^1^ Non-fibre carbohydrate, calculated by 1000-CP-NDF-Fat-Ash.

**Table 2:** PCR primers for qPCR analyses.

Target Species	Primer Sequence (5′)	Gene Bank Accession No.	Size (bp)
Total bacteria	F: CGGCAACGAGCGCAACCC R: CCATTGTAGCACGTGTGTAGCC	CP058023.1	147
Total anaerobic fungi	F: GAGGAAGTAAAAGTCGTAACAAGGTTTC R: CAAATTCACAAAGGGTAGGATGATT	GQ355327.1	120
Total protozoa	F: GCTTTCGWTGGTAGTGTATT R: CTTGCCCTCYAATCGTWCT	HM212038.1	234
Total methanogens	F: TTCGGTGGATCDCARAGRGC R: GBARGTCGWAWCCGTAGAATCC	GQ339873.1	160
*R.* *albus*	F: CCCTAAAAGCAGTCTTAGTTCG R: CCTCCTTGCGGTTAGAACA	CP002403.1	176
*R. flavefaciens*	F: ATTGTCCCAGTTCAGATTGC R: GGCGTCCTCATTGCTGTTAG	AB849343.1	173
*B*. *fibrisolvens*	F: ACCGCATAAGCGCACGGA R: CGGGTCCATCTTGTACCGATAAAT	HQ404372.1	65
*F. succinogenes*	F: GTTCGGAATTACTGGGCGTAAA R: CGCCTGCCCCTGAACTATC	AB275512.1	121
*Rb. amylophilus*	F: CTGGGGAGCTGCCTGAATG R: GCATCTGAATGCGACTGGTTG	MH708240.1	102
*P. ruminicola*	F: GAAAGTCGGATTAATGCTCTATGTTG R: CATCCTATAGCGGTAAACCTTTGG	LT975683.1	74

**Table 3:** Effects of supplementary riboflavin (RF) on dry matter intake (DMI), average daily gain (ADG) and feed conversion ratio (FCR) in Hu lambs.

Item	Treatments *				SEM	*p*-Value		
	Control	LRF	MRF	HRF		Treatment	Linear	Quadratic
DMI (kg d^−1^)	1.78	1.78	1.72	1.66	0.094	0.518	0.148	0.321
Body weight (kg)								
Initial body weight	18.9	18.8	18.8	18.7	0.829	0.995	0.793	0.966
Final body weight	36.6	37.8	38.8	36.7	1.20	0.255	0.726	0.170
ADG (kg d^−1^)	0.31 ^b^	0.33 ^ab^	0.34 ^a^	0.31 ^b^	0.014	0.037	0.447	0.023
FCR (kg kg^−1^)	5.84 ^a^	5.48 ^ab^	4.99 ^b^	5.35 ^ab^	0.255	0.019	0.026	0.061

^a, b^ Different superscript letters in the same variable indicate statistical differences (*p* < 0.05). * Control and low, medium, and high RF (LRF, MRF, HRF) groups were respectively administered 0, 15, 30 and 45 mg/kg DM RF. Data are reported as means (*n* = 48).

**Table 4:** Effects of supplemental riboflavin (RF) intake on nutrient digestibility and rumen fermentation in Hu lambs.

Item	Treatments *				SEM	*p*-Value		
	Control	LRF	MRF	HRF		Treatment	Linear	Quadratic
Digestibility (%)								
Dry matter	64.4 ^b^	66.0 ^ab^	67.8 ^a^	64.8 ^b^	1.04	0.015	0.449	0.016
Organic matter	66.8 ^b^	68.3 ^ab^	70.2 ^a^	67.5 ^b^	1.03	0.022	0.327	0.022
Crude protein	71.8 ^b^	73.6 ^ab^	75.4 ^a^	72.5 ^b^	1.10	0.019	0.345	0.014
Neutral detergent fibre	46.4 ^b^	48.1 ^ab^	51.3 ^a^	47.4 ^b^	1.67	0.045	0.301	0.049
Acid detergent fibre	42.7 ^b^	43.4 ^b^	48.2 ^a^	44.1 ^b^	1.74	0.021	0.165	0.042
Ruminal fermentation								
pH	6.51 ^a^	6.36 ^ab^	6.24 ^b^	6.37 ^ab^	0.080	0.014	0.042	0.068
Total VFA (mM)	106 ^c^	126 ^ab^	134 ^a^	120 ^b^	4.71	0.001	0.005	0.061
Mol/100 mol								
Acetate (A)	64.7 ^b^	67.0 ^a^	68.1 ^a^	66.9 ^a^	0.89	0.002	0.032	0.411
Propionate (P)	22.0 ^a^	19.5 ^b^	19.2 ^b^	20.8 ^b^	0.50	0.030	0.039	0.112
Butyrate	10.00	10.30	9.23	9.36	1.18	0.770	0.421	0.722
Valerate	2.43	2.10	2.28	1.84	0.26	0.140	0.058	0.160
Isobutyrate	0.20 ^c^	0.30 ^b^	0.34 ^a^	0.29 ^b^	0.02	0.001	0.022	0.073
Isovalerate	0.81	0.79	0.83	0.79	0.10	0.974	0.973	0.980
A: P^2^	2.96 ^c^	3.45 ^a^	3.55 ^a^	3.22 ^b^	0.08	0.001	0.043	0.082
Ammonia N (mg 100 mL^−1^)	10.25	9.61	9.00	9.74	0.86	0.551	0.431	0.389

^a,b,c^ Different superscript letters in the same variable indicate statistical differences (*p* < 0.05). * Control and low, medium, and high RF (LRF, MRF, HRF) groups were respectively administered 0, 15, 30 and 45 mg/kg DM RF. Data are reported as means (*n* = 48).

**Table 5:** Effects of supplemental riboflavin (RF) intake on ruminal microbial enzymatic activity and microbial populations in Hu lambs.

Item	Treatments *				SEM	*p*-Value		
	Control	LRF	MRF	HRF		Treatment	Linear	Quadratic
Microbial enzyme activity ^1^								
Carboxymethyl-cellulase	0.32 ^b^	0.36 ^ab^	0.41 ^a^	0.38 ^ab^	0.031	0.041	0.032	0.121
Cellobiase	0.55	0.60	0.63	0.59	0.041	0.276	0.252	0.152
Xylanase	0.89 ^b^	0.96 ^ab^	1.00 ^a^	1.00 ^a^	0.042	0.038	0.008	0.214
Pectinase	1.41 ^b^	1.64 ^ab^	1.86 ^a^	1.61 ^ab^	0.143	0.037	0.011	0.122
α-amylase	2.54	2.70	2.99	2.71	0.260	0.384	0.316	0.271
Protease	1.19 ^b^	1.37 ^ab^	1.52 ^a^	1.39 ^ab^	0.098	0.024	0.046	0.071
Microbiota (copies mL^−1^)								
Total bacteria, × 10 ^12^	2.68 ^b^	3.29 ^ab^	3.96 ^a^	3.21 ^ab^	0.365	0.016	0.047	0.081
Total anaerobic fungi, × 10 ^10^	4.24 ^c^	6.79 ^b^	8.85 ^a^	7.02 ^b^	0.786	0.001	0.002	0.051
Total protozoa, ×10^9^	1.71 ^b^	3.48 ^b^	5.14 ^a^	3.17 ^ab^	0.841	0.004	0.028	0.103
*Methanogens*, ×10^10^	0.67	0.72	0.90	0.78	0.108	0.192	0.135	0.197
*R. albus,* ×10^8^	1.06 ^b^	1.78 ^ab^	2.17 ^a^	1.71 ^ab^	0.350	0.029	0.048	0.111
*R. flavefaciens*, ×10^9^	1.47 ^b^	1.81 ^ab^	2.08 ^a^	1.74 ^ab^	0.200	0.041	0.028	0.122
*F. succinogenes*, ×10^9^	1.65 ^b^	1.95 ^ab^	2.38 ^a^	1.95 ^ab^	0.239	0.040	0.012	0.334
*B. fibrisolvens*, ×10^9^	2.10 ^b^	2.64 ^b^	3.19 ^a^	2.64 ^ab^	0.362	0.046	0.028	0.087
*P. ruminicola*, ×10^10^	1.24 ^b^	1.44 ^ab^	1.84 ^a^	1.59 ^ab^	0.181	0.019	0.022	0.119
*Rb. amylophilus*, ×10^8^	1.41 ^b^	1.65 ^ab^	2.06 ^a^	1.70 ^ab^	0.220	0.049	0.030	0.054

^a,b,c^ Different superscript letters in the same variable indicate statistical differences (*p* < 0.05). * Control and low, medium, and high RF (LRF, MRF, HRF) groups were respectively administered 0, 15, 30, and 45 mg/kg DM RF. Data are reported as means (*n* = 48). ^1^ Enzymatic activity units were as follows: α-amylase (μmol glucose/min per mL), pectinase (μmol D-galacturonic acid/min per mL), carboxymethyl-cellulase (μmol glucose/min per mL), xylanase (μmol xylose/min per mL), cellobiase (μmol glucose/min per mL), protease (μg hydrolyzed protein/min per mL).

**Table 6:** Effects of supplemental riboflavin (RF) intake on urinary purine derivative (PD) excretion and MCP in Hu lambs.

Item	Treatments *				SEM	*p*-Value		
	Control	LRF	MRF	HRF		Treatment	Linear	Quadratic
Allantoin (mmol d^−1^)	8.61 ^b^	9.86 ^b^	12.03 ^a^	8.90 ^b^	0.916	0.012	0.470	0.026
Uric acid (mmol d^−1^)	1.46	1.69	2.09	1.75	0.466	0.616	0.397	0.483
Xanthine + hypoxanthine (mmol d^−1^)	0.82 ^b^	0.96 ^ab^	1.14 ^a^	0.87 ^b^	0.087	0.014	0.362	0.019
TPD^1^ (mmol d^−1^)	10.9 ^b^	12.8 ^ab^	15.3 ^a^	11.6 ^b^	1.16	0.014	0.370	0.019
MCP^2^ (g d^−1^)	58.3 ^b^	68.7 ^ab^	82.4 ^a^	62.4 ^b^	6.44	0.014	0.374	0.020

^a,b,c^ Different superscript letters in the same variable indicate statistical differences (*p* < 0.05). * Control and low, medium, and high RF (LRF, MRF, HRF) groups were respectively administered 0, 15, 30 and 45 mg/kg DM RF. Data are reported as means (*n* = 48). ^1^ TPD, Total purine derivatives, (mmol day^−1^) = Allantoin + Uric acid + Xanthine + hypoxanthine ^2^ MCP was calculated by the following formulas (Chen and Gomes, 1992): Y = 0.84X + (0.150 W^0.75^ e^−0.25 X^). X (mmol/d) = microbial purine absorption, Y (mmol day^−1^) = urinary PD excretion; MCP (g day^−1^) = X ×70 × 6.25/(0.116 × 0.83 × 1000).

## Data Availability

The datasets generated during the current study are available from the corresponding author on reasonable request.
